# Identification of 16S rRNA and Virulence-Associated Genes of *Arcobacter* in Water Samples in the Kathmandu Valley, Nepal

**DOI:** 10.3390/pathogens8030110

**Published:** 2019-07-26

**Authors:** Rajani Ghaju Shrestha, Yasuhiro Tanaka, Jeevan B. Sherchand, Eiji Haramoto

**Affiliations:** 1 Division of Sustainable Energy and Environmental Engineering, Osaka University, Suita, Osaka 565-0871, Japan; 2 Interdisciplinary Center for River Basin Environment, University of Yamanashi, 4-3-11 Takeda, Kofu, Yamanashi 400-8511, Japan; 3 Department of Environmental Sciences, University of Yamanashi, 4-4-37 Takeda, Kofu, Yamanashi 400-8510, Japan; 4 Institute of Medicine, Tribhuvan University Teaching Hospital, Kathmandu 1524, Nepal

**Keywords:** *Arcobacter*, groundwater, Kathmandu Valley, surface water, virulence gene

## Abstract

This study aimed to determine the prevalence of *Arcobacter* and five associated virulence genes (*cadF*, *ciaB*, *mviN*, *pldA*, and *tlyA*) in water samples in the Kathmandu Valley, Nepal. A total of 286 samples were collected from deep tube wells (*n* = 30), rivers (*n* = 14), a pond (*n* = 1), shallow dug wells (*n* = 166), shallow tube wells (*n* = 33), springs (*n* = 21), and stone spouts (*n* = 21) in February and March (dry season) and August (wet season), 2016. Bacterial DNA was extracted from the water samples and subjected to SYBR Green-based quantitative PCR for 16S rRNA and virulence genes of *Arcobacter*. The 16S rRNA gene of *Arcobacter* was detected in 36% (40/112) of samples collected in the dry season, at concentrations ranging from 5.7 to 10.2 log copies/100 mL, and 34% (59/174) of samples collected in the wet season, at concentrations of 5.4–10.8 log copies/100 mL. No significant difference in *Arcobacter* 16S rRNA gene-positive results was observed between samples collected in the two seasons (*p* > 0.05). Seventeen (17%), 84 (84%), 19 (19%), 23 (23%), and 17 (17%) of the 99 *Arcobacter* 16S rRNA gene-positive samples were also positive for *cadF*, *ciaB*, *mviN*, *pldA*, and *tlyA*, respectively. At least one virulence gene was detected in 87 (88%) of the 99 *Arcobacter* 16S rRNA gene-positive samples. The presence of *Arcobacter* and the virulence genes in these samples illustrates the persistence of pathogenic bacteria in the environment and highlights the importance of regular monitoring of water for pathogens.

## 1. Introduction

Fecal contamination of water sources has been a problem in developing countries and is a major factor in the spread of waterborne diseases. Nepal is facing extensive contamination of environmental water sources where enteric pathogens, such as bacteria, viruses, and protozoa, are often detected [1,2,3,4,5,6,7,8,9,10]. Fecal contamination mainly occurs through leakage of sewage pipes, improper disposal of feces, poor construction design of septic tanks, and the use of animal manure in agricultural fields [11,12].

*Arcobacter* has been detected in the groundwater and river water of many countries including Czech Republic [13], Japan [14], South Africa [15], Spain [16,17], Turkey [18,19,20], and USA [21]. This global prevalence of *Arcobacter* has had a major impact on groundwater and surface water in the Kathmandu Valley [1,2]. *Arcobacter* was one of the dominant pathogenic bacteria isolated from groundwater and surface water samples analyzed by next-generation sequencing [1,2]. Moreover, a recently developed SYBR Green-based quantitative PCR (qPCR) assay, which is highly specific to most of the known species of *Arcobacter*, succeeded in detecting the 16S rRNA gene of *Arcobacter* in 27% (13/48) of the water samples analyzed [2]. Previous studies conducted in the Kathmandu Valley were limited to small numbers of water samples collected in a single season, which does not allow seasonal variations of *Arcobacter* within the valley to be identified [1,2].

The pathogenicity of an organism is characterized according to the presence of virulence genes. Miller et al. (2007) identified several virulence genes, such as *cadF* and *cjl349* (fibronectin binding proteins), which are homologs of the putative virulence determinants present in *Campylobacter jejuni* [22]. Similarly, *ciaB* (invasion protein), *mviN* (a protein required for peptidoglycan biosynthesis), *pldA* (phospholipase), and *tlyA* (hemolysin) are homologs of putative virulence determinants of *Campylobacter*, *irgA* (iron-regulated outer membrane protein) and *iroE* (siderophore esterase) are homologs found in uropathogenic *Escherichia coli*, and *hecA* (filamentous hemagglutinin) and *hecB* (hemolysin activation protein) are present in *Erwinia crysthanthemi* contributing to epidermal cell killing [22,23,24,25,26]. Although it remains unclear whether these virulence genes have similar functions to their homologs in different pathogenic bacteria, a study by Levican et al. (2013) showed little or no invasion of cells by *Arcobacter* strains that lack virulence genes [27]. Therefore, the detection and quantification of virulence genes of *Arcobacter* may elucidate its role in waterborne infections. 

Based on these factors, the aim of this study was to investigate the prevalence of 16S rRNA and five virulence-associated genes (*cadF*, *ciaB*, *mviN*, *pldA*, and *tlyA*) of *Arcobacter* in water samples collected from different sites of the Kathmandu Valley in both the dry and the wet season. These five genes were selected based on their high presence in *Arcobacter* isolates obtained from various types of fecal, food, and environmental samples [28,29,30,31].

## 2. Results

### 2.1. Detection of 16S rRNA Gene of Arcobacter in Water Samples

A total of 112 water samples were collected from different sources of water in the dry season of 2016. As shown in Table 1, 16S rRNA gene of *Arcobacter* was detected in 20% (2/5), 100% (1/1), 100% (1/1), 41% (32/79), 9% (1/11), 13% (1/8), and 29% (2/7) of deep tube well, pond, river, shallow dug well, shallow tube well, spring, and stone spout water samples, respectively. The concentration of *Arcobacter* 16S rRNA gene was highest in the river water sample with 10.2 log copies/100 mL, followed by deep tube wells (7.6 ± 0.4 log copies/100 mL) and shallow dug wells (6.2 ± 0.8 log copies/100 mL). Among 174 water samples collected in the wet season of 2016, 16S rRNA gene of *Arcobacter* was detected in 12% (3/25), 92% (12/13), 43% (37/87), 9% (2/22), 23% (3/13), and 14% (2/14) of deep tube well, river, shallow dug well, shallow tube well, spring, and stone spout water samples, respectively. The highest concentration of 16S rRNA gene of *Arcobacter* was detected in the river water (9.3 ± 1.3 log copies/100 mL), followed by deep tube wells (7.1 ± 0.5 log copies/100 mL) and stone spouts (6.4 ± 0.4 log copies/100 mL). A significant difference in *Arcobacter*-positive results was not observed between samples collected in the dry and wet season (*p* > 0.05).

Water sampling was conducted in both seasons at 84 locations, with 29 (35%) and 33 (39%) samples testing positive for 16S rRNA gene of *Arcobacter* in the dry and wet season, respectively. A significant difference in the concentrations of 16S rRNA gene of *Arcobacter* was not evident between water samples collected at the same location but in different seasons (*p* > 0.05). Sixteen (19%) samples (one stone spout and 15 shallow dug wells) were positive for 16S rRNA gene of *Arcobacter* regardless of the sampling season. The samples were also divided into two land-use categories (agricultural or built-up areas) [8]. In the dry season, *Arcobacter* 16S rRNA gene was detected in 35% (16/46) and 36% (24/66) of the samples collected in agricultural and built-up areas, respectively. In contrast, the bacterium was detected in 41% (24/59) and 30% (35/115) of samples collected in agricultural and built-up areas during the wet season, respectively. There was no significant difference in the detection ratios of *Arcobacter* between agricultural and built-up areas (*p* > 0.05).

### 2.2. Detection of Arcobacter Virulence Genes in Water Samples

A total of five virulence genes, namely *cadF*, *ciaB*, *mviN*, *pldA*, and *tlyA*, were tested in *Arcobacter* qPCR-positive water samples. In the dry season, *cadF*, *ciaB*, *mviN*, *pldA*, and *tlyA* genes were detected in 1 (3%), 35 (88%), 2 (5%), 8 (20%), and 2 (5%) of 40 *Arcobacter*-positive water samples, respectively (Table 2). The river water sample was the only sample, where all five virulence genes were detected with the highest concentrations (8.4–10.1 copies/100 mL, depending on genes). The number of samples positive for at least one virulence gene was 35.

In the wet season, the number of *Arcobacter* qPCR-positive samples was 59, of which 16 (27%), 49 (83%), 17 (29%), 15 (25%), and 15 (25%) were positive for *cadF*, *ciaB*, *mviN*, *pldA*, and *tlyA*, respectively (Table 3). In total, seven river water and one shallow dug well water samples contained all five virulence genes, and 52 *Arcobacter* qPCR-positive samples were positive for at least one of the genes. The highest mean concentrations of all five virulence genes was observed in the river water samples (8.6–9.4 log copies/100 mL).

## 3. Discussion

In this study, 35% (99/286) of water samples tested were contaminated with 16S rRNA gene of *Arcobacter*. In our previous study, 16S rRNA gene of *Arcobacter* was detected in shallow dug wells (39%, 11/28), a deep tube well (20%, 1/5), and a river water sample (100%, 1/1), but not in shallow tube wells, stone spouts, and springs [2]. In the Kathmandu Valley, the limited distribution of drinking water from Kathmandu Upatyaka Khanepani Limited (115 and 69 million liters per day (MLD) in wet and dry seasons, respectively), and high demand for drinking water (370 MLD) has compelled residents to use groundwater and surface water for many household chores [32]. Even though groundwater and surface water are mostly used for bathing, laundry, cleaning, gardening, and other purposes, 12% and 22% of people use groundwater for drinking and cooking, respectively [33]. Apart from piped water and groundwater, the residents use jar and tanker water for drinking. However, these sources of water do not meet the World Health Organization guideline values for safe drinking water [34,35]. Only 10.2%, 9.6%, and 3.7% of people use treated water for washing vegetables, brushing teeth, and bathing purposes [33]. As such, people living in the valley are at high risk of contracting waterborne diseases due to the contamination of groundwater and surface water with *Arcobacter*.

*Arcobacter butzleri*, *Arcobacter skirrowii*, and *Arcobacter cryaerophilus* are commonly detected in the surface water and groundwater of Japan (23%, 4/17), South Africa (55%, 6/11), Spain (59%, 17/29), and Turkey (41%, 17/41) [14,15,16,19]. Other species of *Arcobacter* beside *A. butzleri*, *A. skirrowii*, and *A. cryaerophilus* also exhibit pathogenic properties, such as adhesion, invasion, and cytotoxicity [27,36]. The findings of our study expand the knowledge of the prevalence of *Arcobacter* in surface water and groundwater samples, especially as we targeted a wide range of 16S rRNA genes of *Arcobacter* species along with large numbers of samples, collected in two different seasons. The presence of *Arcobacter* has been linked to waterborne and foodborne diseases, with water being one of the important routes of transmission [16,37,38]. Therefore, groundwater and surface water contaminated with *Arcobacter* consumed may cause waterborne and foodborne diseases. *Arcobacter* densities were found to be higher in diarrheal than in non-diarrheal stools [39]. Diarrhea remains one of the leading causes of morbidity and mortality in Nepal, and diarrheal samples are often diagnosed with enteric protozoa, bacteria, and even viruses in clinical laboratories [40,41,42,43]. To date, the prevalence of *Arcobacter* in diarrheal stool samples in Nepal has not been studied. As an emerging pathogen, the determination of the prevalence of *Arcobacter* in water and stool samples must become a priority. 

Among the 286 water samples tested in this study, 250 (shallow dug well, *n* = 166; shallow tube well, *n* = 33; deep tube wells, *n* = 30; and stone spouts, *n* = 21) were previously used for the identification of human and animal fecal contamination using host-associated Bacteroidales genetic markers [8]. Fifty-five (22%), 28 (11%), and 8 (3%) of the water samples were found to contain human, ruminant, and pig fecal markers, respectively [8], whereas 16S rRNA gene of *Arcobacter* was detected more frequently in 81 (32%) water samples. If the source of *Arcobacter* contamination is confirmed as human or animal feces, *Arcobacter* should be considered for the identification of human and animal fecal contamination. Thus, further studies are required to evaluate the feasibility of using *Arcobacter* to identify sources of fecal contamination of water, based on the genetic diversity of *Arcobacter* spp.

As summarized in Table 4, a positive correlation has been demonstrated between the concentration of *E. coli* [8] and the positive ratios of *Arcobacter* in the water samples tested. As the concentrations of *E. coli* increased, the percentages of *Arcobacter* 16S rRNA gene-positive samples also increased. There were two samples that were positive for *Arcobacter* 16S rRNA gene but were *E. coli*–negative. This result is supported by the study of Malla et al. (2018), in which 23% (13/56) of *E. coli*–negative samples were found to be positive for at least one human or ruminant fecal marker [8].

Among the five virulence-associated genes tested, *ciaB* was the most prevalent in both the dry (88%, 35/40) and wet (83%, 49/59) season. This agrees with previous studies in which the gene was detected in all strains of *A. butzleri*, *A. cryaerophilus*, and *A. skirrowii* isolated from water samples [13,44]. In *Arcobacter*-positive samples from food, cattle and sheep feces, chicken carcasses, processing equipment of slaughterhouses, animals, and humans, *ciaB* was the most dominant virulence-associated gene [28,29,44]. Other prevalent virulence genes are *pldA* (20%, 8/40) and *mviN* (29%, 17/59), which were detected in samples collected in the dry and wet season, respectively. The *pldA*, *mviN*, *cj1349*, and *tlyA* genes were detected in 100% of *A. butzleri* strains isolated from water samples, and the *mviN* gene was detected in 100% of *A. cryaerophilus* isolates [13]. The high detection rates of these genes may be due to their specificity for *A. butzleri* and *A. cryaerophilus* isolates. In our study, several other species of *Arcobacter* including *A. butzleri* and *A. cryaerophilus* were targeted. Only a few studies have been published on the detection of these virulence genes in water samples [13,44]. This highlights the importance of our study in assessing the prevalence of these genes in a large number of water samples, through screening for the majority of the known *Arcobacter* species.

*A. butzleri*, *A. cryaerophilus*, and *A. skirrowii* are known pathogenic species of *Arcobacter* responsible for diarrhea and foodborne infection [16,39]. Virulence-associated genes have been identified in strains of these three pathogens isolated from various animals; clinical samples; and food, fecal, and water samples. As virulence genes are associated with the pathogenic characteristics of *Arcobacter*, such as adhesion, invasion, and cytotoxicity in different cell lines, the detection of these genes in surface water and groundwater needs to be taken seriously [27,36]. All the genes tested were present in several individual samples, especially those from river water and a shallow dug well. Thus, the detection of virulence-associated genes along with *Arcobacter* in water samples may represent a high risk of waterborne disease transmission.

In conclusion, this study demonstrates the high presence of 16S rRNA and five virulence-associated genes of *Arcobacter* in water samples collected in the Kathmandu Valley. We also highlighted differences in the detection ratios of *Arcobacter* between the dry and wet season, and agricultural and built-up areas. The presence of pathogenic bacteria in groundwater and surface water underlines the importance of regular monitoring for pathogens and rapid identification of contamination sources.

## 4. Materials and Methods

### 4.1. Collection of Water Samples

In this study, a total of 286 water samples were collected in the Kathmandu Valley in 2016. One hundred two water samples were collected in February and March 2016 (dry season) from deep tube wells (*n* = 5), shallow dug wells (*n* = 79), shallow tube wells (*n* = 11), and stone spouts (*n* = 7), whereas 148 water samples were collected in August 2016 (wet season) from deep tube wells (*n* = 25), shallow dug wells (*n* = 87), shallow tube wells (*n* = 22), and stone spouts (*n* = 14), as described previously [8]. Water samples were also collected from a pond (*n* = 1), a river (*n* = 1), and springs (*n* = 8) in February and March 2016, and from rivers (*n* = 13) and springs (*n* = 13) in August 2016. Two hundred milliliter samples were collected in autoclaved 100 mL plastic bottles, which were stored in an ice bag and transported to the laboratory within 4 h of collection [8].

### 4.2. Bacterial DNA Extraction

Water samples (100 mL of groundwater and 10 mL of river water) were filtered using a disposable filter unit preset with a nitrocellulose membrane (pore size, 0.22 µm; diameter, 47 mm; Nalgene, Tokyo, Japan) as described previously [8]. Bacterial DNA was extracted from the membrane filter using a Cica Geneus DNA Extraction Reagent (Kanto Chemical, Tokyo, Japan). In brief, 5 mL of Tris-EDTA buffer (pH 7.4) was added to a 50 mL tube containing the membrane filter, followed by shaking and vortex mixing. Finally, 300 µL of DNA was extracted from 160 µL of the mixture solution with 20 µL of Buffer A and 200 µL of Buffer B.

### 4.3. qPCR of 16S rRNA Gene of Arcobacter and Its Virulence Genes

For the quantification of 16S rRNA gene of *Arcobacter*, qPCR was performed using 2 µL of template DNA, 12.5 µL of a MightyAmp for Real Time (SYBR Plus) (Takara Bio, Kusatsu, Japan), 0.1 µL of 1 µM Arco-F and Arco-R-rev primers targeting 16S rRNA gene of the genus [2], and 10.3 µL of ultrapure water. The quantification of virulence genes (*ciaB*, *cadF*, *mviN*, *pldA*, and *tlyA*) was performed on *Arcobacter* qPCR-positive water samples. In brief, 2 µL of template DNA, 12.5 µL of a SYBR Premix Ex Taq II (Tli RNaseH Plus) (Takara Bio), 0.1 µL of 1 µM forward and reverse primers [28], and 10.3 µL of ultrapure water were used. The thermal conditions were as follows: For 16S rRNA gene of *Arcobacter*, 98 °C for 2 min, followed by 35 cycles of 98 °C for 10 s, 55 °C for 30 s, and 68 °C for 40 s; for the *ciaB* and *tlyA* gene, 95 °C for 30 s, followed by 35 cycles of 94 °C for 15 s, 55 °C for 30 s, and 72 °C for 20 s; and for *cadF*, *mviN*, and *pldA* genes, 95 °C for 30 s, followed by 35 cycles of 94 °C for 15 s, 56 °C for 45 s, and 72 °C for 20 s, using the Thermal Cycler Dice Real Time System Single TP850 (Takara Bio). Following amplification, thermal conditions of 95 °C for 15 s, 60 °C for 30 s, and 95 °C for 15 s were used to identify a single peak melting temperature. A single melting temperature peak of approximately 85, 79, 80, 78, 79, and 77 °C was considered to be derived from amplified 16S rRNA gene of *Arcobacter*, *cadF*, *ciaB*, *mviN*, *pldA*, and *tlyA* genes, respectively.

### 4.4. Statistical Analysis

Chi-square testing was performed using Microsoft Excel 2016 (Microsoft Corporation, Redmond, WA, USA) to determine the difference in positive ratios of 16S rRNA gene of *Arcobacter* relative to concentrations of *E. coli*. This test was also used to confirm significant differences in the distribution of 16S rRNA gene of *Arcobacter* through different land covers and seasons. Wilcoxon’s signed rank test was performed to identify differences in the concentrations of 16S rRNA gene of *Arcobacter* in water samples collected at the same location but in different seasons. Differences were considered statistically significant if the resulting *p* value was <0.05.

## Figures and Tables

**Table 1:** Detection of 16S rRNA gene of *Arcobacter* in water samples.

Sources of Water	February–March 2016 (Dry Season)		August 2016 (Wet Season)	
	No. of Positive Samples/No. of Tested Samples (% Positive)	Concentration *^a^* (Mean ± SD *^b^*)	No. of Positive Samples/No. of Tested Samples (% Positive)	Concentration (Mean ± SD)
Deep tube well	2/5 (20)	7.6 ± 0.4	3/25 (12)	7.1 ± 0.5
Pond	1/1 (100)	6.0	– *^c^*	–
River	1/1 (100)	10.2	12/13 (92)	9.3 ± 1.3
Shallow dug well	32/79 (41)	6.2 ± 0.8	37/87 (43)	6.0 ± 0.6
Shallow tube well	1/11(9)	7.1	2/22 (9)	5.6 ± 0.1
Spring	1/8 (13)	5.7	3/13 (23)	6.0 ± 0.2
Stone spout	2/7 (29)	5.9	2/14 (14)	6.4 ± 0.4
Total	40/112 (36)		59/174 (34)	

*^a^* Concentrations among positive samples in log copies/100 mL; *^b^* SD, standard deviation; *^c^* not sampled.

**Table 2:** Detection of virulence genes of *Arcobacter* in water samples collected in February–March 2016 (dry season).

Sources of Water	No. of Samples Positive for *Arcobacter* 16S rRNA Gene	No. of Positive Samples (Concentration *^a^* (Mean ± SD *^b^*))				
		*cadF*	*ciaB*	*mviN*	*pldA*	*tlyA*
Deep tube well	2	0	2 (7.5 ± 0.3)	0	1 (8.1)	0
Pond	1	0	1 (6.2)	0	0	0
River	1	1 (9.0)	1 (10.1)	1 (9.0)	1 (9.0)	1 (8.4)
Shallow dug well	32	0	27 (6.5 ± 0.7)	1 (6.9)	5 (7.8 ± 0.7)	1 (6.3)
Shallow tube well	1	0	1 (7.0)	0	0	0
Spring	1	0	1 (5.9)	0	0	0
Stone spout	2	0	2 (5.8 ± 0.1)	0	1 (8.2)	0
Total (% Positive)	40	1 (3%)	35 (88%)	2 (5%)	8 (20%)	2 (5%)

*^a^* Concentrations among positive samples in log copies/100 mL; *^b^* SD, standard deviation.

**Table 3:** Detection of virulence genes of *Arcobacter* in water samples collected in August 2016 (wet season).

Sources of Water	No. of Samples Positive for *Arcobacter* 16S rRNA Gene	No. of Positive Samples (Concentration *^a^* (Mean ± SD *^b^*))				
		*cadF*	*ciaB*	*mviN*	*pldA*	*tlyA*
Deep tube well	3	1 (5.8)	3 (6.8 ± 0.6)	0	0	1 (6.5)
River	12	8 (9.2 ± 0.4)	11 (9.4 ± 1.1)	10 (9.3 ± 0.6)	8 (9.1 ± 0.5)	9 (8.6 ± 0.5)
Shallow dug well	37	7 (6.3 ± 0.7)	31 (6.3 ± 0.6)	7 (7.5 ± 0.5)	5 (6.3 ± 0.7)	5 (6.7 ± 0.5)
Shallow tube well	2	0	0	0	1 (7.4)	0
Spring	3	0	3 (6.1 ± 0.4)	0	0	0
Stone spout	2	0	1 (6.7)	0	1 (8.6)	0
Total	59	16 (27%)	49 (83%)	17 (29%)	15 (25%)	15 (15%)

*^a^* Concentrations among positive samples in log copies/100 mL; *^b^* SD, standard deviation.

**Table 4:** Relationship between *Arcobacter* and *Escherichia coli* in water samples.

Concentration of *E. coli ^a^* (log Most Probable Number/100 mL)	No. of Samples	No. of Samples Positive for *Arcobacter* 16S rRNA Gene (% Positive)
<0	58	2 (2%)
0–1.0	45	10 (10%)
1.1–2.0	69	21 (21%)
2.1–3.0	53	26 (26%)
3.1–4.0	35	16 (16%)
>4.0	26	24 (24%)
Total	286	99 (35%)

*^a^* Data from Malla et al. (2018) [8].
